# *In Vitro* and *In Vivo* Toxicity Profiling of Ammonium-Based Deep Eutectic Solvents

**DOI:** 10.1371/journal.pone.0117934

**Published:** 2015-02-13

**Authors:** Maan Hayyan, Chung Yeng Looi, Adeeb Hayyan, Won Fen Wong, Mohd Ali Hashim

**Affiliations:** 1 Department of Civil Engineering, University of Malaya, Kuala Lumpur, Malaysia; 2 University of Malaya Centre for Ionic Liquids (UMCiL), University of Malaya, Kuala Lumpur, Malaysia; 3 Department of Pharmacology, Faculty of Medicine, University of Malaya, Kuala Lumpur, Malaysia; 4 Department of Chemical Engineering, University of Malaya, Kuala Lumpur, Malaysia; 5 Department of Medical Microbiology, Faculty of Medicine, University of Malaya, Kuala Lumpur, Malaysia; Second University of Naples, ITALY

## Abstract

The cytotoxic potential of ammonium-based deep eutectic solvents (DESs) with four hydrogen bond donors, namely glycerine (Gl), ethylene glycol (EG), triethylene glycol (TEG) and urea (U) were investigated. The toxicity of DESs was examined using *In Vitro* cell lines and *In Vivo* animal model. IC_50_ and selectivity index were determined for the DESs, their individual components and their combinations as aqueous solutions for comparison purposes. The cytotoxicity effect of DESs varied depending on cell lines. The IC_50_ for the Gl_DES,_ EG_DES,_ U_DES_ and TEG_DES_ followed the sequence of TEG_DES_< Gl_DES_< EG_DES_< U_DES_ for OKF6, MCF-7, A375, HT29 and H413, respectively. Gl_DES_ was selective against MCF-7 and A375, EG_DES_ was selective against MCF-7, PC3, HepG2 and HT29, U_DES_ was selective against MCF-7, PC3, HepG2 and HT29, and TEG_DES_ was selective against MCF-7 and A375. However, acute toxicity studies using ICR mice showed that these DESs were relatively toxic in comparison to their individual components. DES did not cause DNA damage, but it could enhance ROS production and induce apoptosis in treated cancer cells as evidenced by marked LDH release. Furthermore, the examined DESs showed less cytotoxicity compared with ionic liquids. To the best of our knowledge, this is the first time that combined *In Vitro* and *In Vivo* toxicity profiles of DESs were being demonstrated, raising the toxicity issue of these neoteric mixtures and their potential applicability to be used for therapeutic purposes.

## Introduction

Development of new green solvents is one of the key subjects in green chemistry, and considerable attention has been devoted to the use of ionic liquids (ILs) and DESs to replace the harsh organic solvents currently employed in many chemical processes such as separation, extraction and synthesis [1]. Although it is still unclear whether DESs can be formally classified as ILs, as they contain a substantial portion of molecular components, they possess many of the same attractive solvent properties as regular ILs [2]. DES is a mixture of two or more compounds that has a melting point lower than that of either of its components [3]. This significant depression of the freezing point stems from an interaction between the halide anion of the salt and the HBD component [3,4].

There remain limitations to the employment of ILs in industrial sectors due to the high cost of synthesis and toxicity to humans and the environment [1]. In contrast, DESs are considered potential environmentally benign solvents for many chemical and industrial applications [5,6]. Due to their unusual characteristics, the possibility of using DESs for different applications has been extensively explored [4,7,8]. Industrial applications of DESs are very promising [9]. There are many advantages for using DESs in industrial applications. They are simple to synthesize since the components (i.e. salt and hydrogen bond donor (HBD)/complexing agent) can be easily mixed and converted to DES without the need for further purification; they have low production cost due to the low cost of raw materials; and DES is expected to exhibit good biocompatibility when using quaternary ammonium salts such as choline chloride (ChCl) [6,10,11].

To implement DESs in industrial applications, the investigation of toxicology profile is indispensable for the assessment of safety, health and environmental impacts. Nevertheless, DESs have not yet been studied and the toxicity data are sparse. Therefore, the cytotoxicity and toxicity of DESs are fundamental aspects that must be addressed before applying DES to industrial applications [12]. Furthermore, since none of these DESs have been registered, their general use as solvents may be restricted because it has been claimed, on the basis of the properties of individual components of DESs, that DESs are non-toxic, eco-friendly, biodegradable and benign solvents [10,11,13–15].

Recently, we investigated the toxicity and cytotoxicity of DESs based on ammonium and phosphonium salts [6,12]. The cytotoxicity effect was tested using brine shrimp (*Artemia salina*) while the toxicity was investigated using the two Gram positive bacteria *Bacillus subtilis* and *Staphylococcus aureus*, and two Gram negative bacteria *Escherichia coli* and *Pseudomonas aeruginosa*. The cytotoxicity of tested DESs was much higher than that of their individual components, indicating that their toxicological behavior was different. It was also found that there was a toxic effect on the studied bacteria for phosphonium-based DESs, indicating their potential application as anti-bacterial agents, while no toxicity effect was observed for ammonium-based DESs. The aim of the recent EU Regulation REACH (EC1907/2006; Registration, Evaluation, Authorization and Restriction of Chemical substances) is to improve the protection of the environment and human health through better and earlier identification of the intrinsic and potentially toxic properties of chemical substances. A key aspect of REACH regulation is the progressive substitution of the most dangerous chemicals with suitable alternatives. At the same time, in order to reduce the number of tests with animals, REACH regulation strongly encourages the use of alternative approaches, such as in vitro methods at cellular and sub-cellular level [16]. If there is a therapeutic response then the major advantage of DESs would be in varying their constituents and molar ratios to improve their pharmacological properties for desired therapeutic applications.

**Fig 1:**
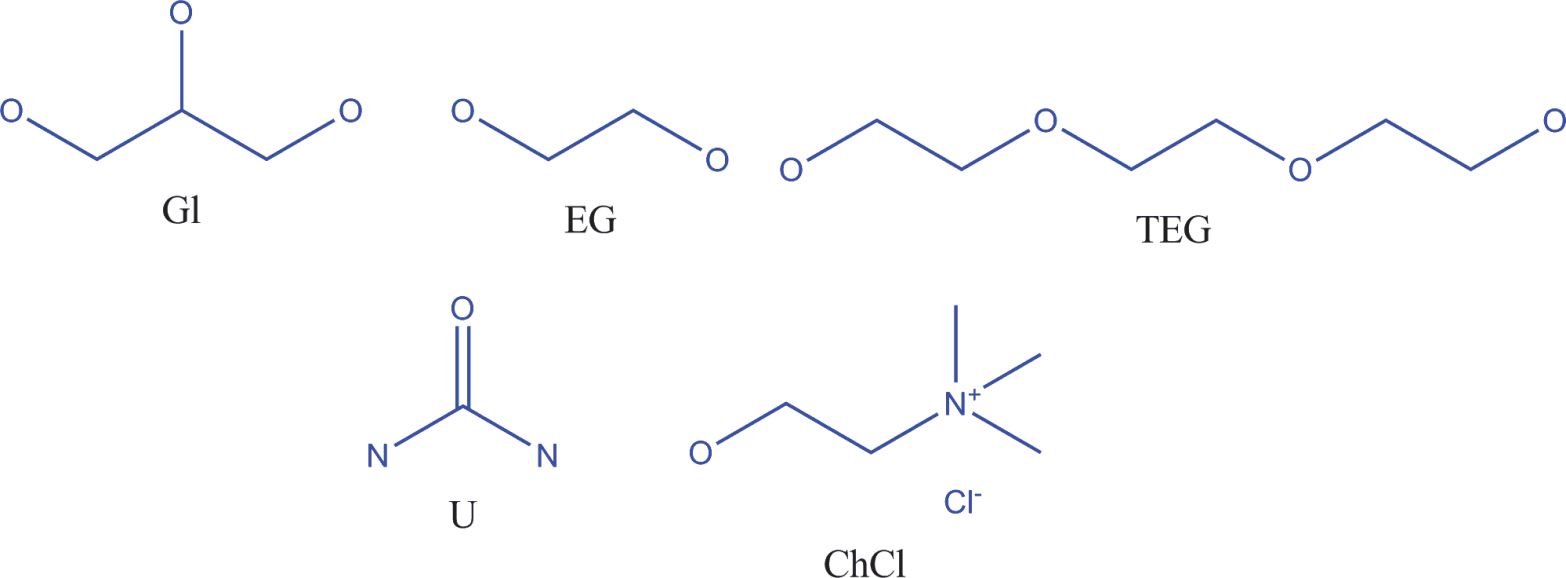
Structures of the salt and HBDs.

In this work, the cytotoxicity of selected ammonium-based DES towards five human cancer cell lines and one normal cell line was investigated. The DESs are based on ChCl combined with four HBDs, namely glycerine (Gl), ethylene glycol (EG), triethylene glycol (TEG) and urea (U) (Fig. 1).

## Materials and Experimental Methodologies

### Synthesis of DES

To prepare the DESs used in this work ChCl (Merck 99%) was dried under vacuum and mixed with the HBD (Merck) at mole ratio 1:3 ChCl to HBD. The mixture was stirred at 300 rpm and 80°C until a homogenous transparent liquid was formed.

### Cell Culture

Human prostate cancer cell line (PC3), human malignant melanoma cell line (A375), human liver hepatocellular cell line (HepG2) and human colon adenocarcinoma cell line (HT29) were purchased from American Type Culture Collection (ATCC, Manassas, VA). The human breast cancer cell line (MCF-7) was acquired from Cell Lines Service (300273; Eppelheim, Germany), human oral keratinocyte cell line (OKF6) cells [17] and carcinoma-derived human oral keratinocyte cells (H413) (Sigma-aldrich, Saint Louis, MO) were received from Professor Ian Charles Paterson. MCF-7, A375, HT29 and HepG2 cells were grown in Dulbecco’s Modified Eagle Medium (DMEM, Life Technologies, Inc., Rockville, MD) supplemented with 10% heat-inactivated fetal bovine serum (FBS, Sigma-Aldrich, St. Louis, MO), 1% penicillin and streptomycin. PC3 cells were grown in Roswell Park Memorial Institute medium (RPMI) supplemented with 10% heat-inactivated fetal bovine serum (FBS, Sigma-Aldrich, St. Louis, MO), 1% penicillin and streptomycin. OKF6 cells were grown in Keratinocyte Serum-Free Medium (KSFM), supplemented with 10% heat-inactivated fetal bovine serum (FBS, Sigma-Aldrich, St. Louis, MO), 1% penicillin and streptomycin. H413 cells were grown in Dulbecco's Modified Eagle Medium/Ham's F-12 (DMEM/F12) supplemented with 10% heat-inactivated fetal bovine serum (FBS, Sigma-Aldrich, St. Louis, MO), 1% penicillin and streptomycin. Cells were cultured in tissue culture flasks (Corning, USA) and were kept in an incubator at 37°C in a humidified atmosphere with 5% CO_2_. For experimental purposes, cells in exponential growth phase (approximately 70–80% confluency) were used.

### MTT Cell Viability Assay

The influence of the solvents was determined by MTT assay (Mosmann, 1983). MCF-7, A375, HT29, HepG2, PC3, OKF6 and H413 cells were treated for 48 h. On the first day, 1.0 × 10^4^ cells were seeded into a 96-well plate for 24 h incubation assay. The cells were incubated overnight at 37°C in 5% CO_2_. On the next day, the cells were treated with a two-fold dilution series of six concentrations of the solvents, and then incubated at 37°C in 5% CO_2_ for 48 h. MTT solution (4,5-dimethylthiazol-2-yl-2,5-diphenyltet​razoliumbromide) was added at 2 mg/mL, and after 2 h of incubation at 37°C in 5% CO_2_, DMSO was added to dissolve the formazan crystals. The plates were then read in Chameleon multitechnology microplate reader (Hidex, Turku, Finland) at 570 nm absorbance. The cell viability percentage after exposure to the solvents for 48 h was calculated by the previously described method [18]. The ratio of the absorbance of treated cells to the absorbance of DMSO-treated control cells was determined as cell viability (percentage). The concentration of the solvents that is required to reduce the absorbance of treated cells to 50% of the DMSO-treated control cells was defined as IC_50_.

### DNA preparation

For DNA preparation, treated and untreated cells were trypsinized and pelleted in a 15 mL tube by centrifugation at 1000 rpm for 2 min. DNA extraction was carried out using DNeasy Blood & Tissue Kit (QIAGEN, Hilden, Germany). In brief, 200 μL lysis buffer (Buffer AL) was added to the cell pellet followed by 10 min incubation at 56°C. Then the lysate was thoroughly mixed with 200 μL ethanol (96–100%), transferred to a DNeasy Mini spin column placed in a 2 mL collection tube and centrifuged at ≥8000 rpm for 1 min. The flow-through was discarded and the spin column was washed twice using AW1 and AW2 buffers before eluting the DNA by adding elution buffer (Buffer AE) and centrifugation at ≥8000 rpm for one min.

### DNA Fragmentation Analysis by Agarose Gel Electrophoresis

Thirty μL of the extracted DNA were mixed with 3 mL loading buffer. The samples were resolved on a 1.2% agarose gel and visualized by UV light after standard ethidium bromide staining.

### Experimental Animals

The acute toxicity of the compounds was evaluated using six Imprinting Control Region (ICR) mice per groups at 8 to 12 weeks of age, with an average body weight of 25.6 g. The animals were assigned equally into 4 groups labeled as vehicle (dH_2_O), high dose (20 g/kg), medium dose (10 g/kg) and low dose (5 g/kg) of the compounds. Prior to administration, the animals were fasted (food but not water) overnight and for 3 to 4 h after compounds administration to eliminate any food inside the gastrointestinal tract that might complicate absorption of the test substance. The animals were observed at 30 min, 2, 4, 24 and 48 h after administration for the onset of clinical or toxicological symptoms as well as mortality and behavioral changes in the mice following the treatment. Animals were euthanized by CO_2_ asphyxiation on 15^th^ day, and serum biochemical and histological (liver and kidney) parameters were determined following the standard method. The mortality of the mice were recorded within 14 days and used to calculate the LD_50_ for each compound. The mice used were housed in specific pathogen free facility at the University of Malaya. This work was designed to minimize animal suffering and number of animals used, and has been approved by the Faculty of Medicine Animal Care and Use Committee (FOMIACUC, Approval No: 2014-05-07/PHAR/R/CYL) at University of Malaya. The work was reported according to ARRIVE guideline.

### ORAC—Antioxidant Activity Assay

**Chemicals.** Fluorescein sodium salt, AAPH (2,2′-Azobis(2-methylpropionamidine) dihydrochloride), quercetin dehydrate and trolox ((±)-6-Hydroxy-2,5,7,8-tetramethylchromane-2-carboxylic acid) were purchased from Sigma-Aldrich.

**ORAC assay.** ORAC (oxygen radical antioxidant capacity) assay was done based on procedures described previously with slight modifications [19]. Compounds were diluted to a final concentration of 100μg/mL, with total reaction volume of 200μL. The assay was performed in a 96-well black microplate, with 25μL of samples, standard (trolox), blank (solvent/PBS) or positive control (quercetin). Subsequently, 150μL of working fluorescein solution was added to each well of assay plate. The plate was incubated at 37°C for at least 5 min. 25μL of AAPH working solution was then added to the wells, making up a total volume of 200μL. Fluorescence was recorded with excitation wavelength of 485nm and emission wavelength of 538nm. Data were collected every 2 min for a duration of 2 h, and were analyzed by calculating the differences between the samples and blank of the area under fluorescence decay curve (AUC). The values were expressed as trolox equivalent (TE).

### Apoptosis assay

Cells pretreated with solvents were harvested and stained with FITC-conjugated annexin V and propidium iodide from apoptosis kit (BD Biosciences, San Jose, CA) as previously described [20]. Percentages of apoptotic cells were measured in a BD FACSCanto II flow cytometer machine (BD Biosciences).

### LDH release assay

Cells were pretreated with different concentrations of the solvents for 48 h and the supernatant of the treated and untreated cells was used to assess the LDH activity. The amount of formazan salt formed was measured in a colorimetric assay as described previously [21]. Intensity of red color formed in the treated and untreated samples is proportional to the LDH activity and to the number of damaged cells. The positive control Triton X-100 was used, at a concentration of 2%, to completely lyse the cells, and DHE release level of the treated cells was expressed as percentage of positive control.

### Cell membrane permeability assay

1×10^4^ cells per well were seeded onto 96-well plate for 16 h. Next, cells were treated with DESs (IC_50_ dosage) and further incubated for 24 h. To examine plasma membrane permeability, cell permeability dye (Excitation 491/Emission 509) were added to live cells and incubated for 1 h, as previously described [22]. Cells were washed three times with PBS before fixing with 4% formaldehyde for 15 min. Nucleus was stained with Hoechst 33258. Cells were visualized and images were captured using Cellomics ArrayScan HCS reader (Thermo Scientific).

### Reactive oxygen species (ROS) assay

ROS assay was carried out to determine the influence of solvents on the production of ROS level in treated MCF-7 cells. 1×10^4^ cells per well were seeded onto 96-well plate and incubated overnight at 37°C with 5% CO_2_. The cells were then treated with different concentrations of the solvents for 24 h and then dihydroethidium (DHE) dye was added into live culture for 30 min. Cells were fixed and washed with wash buffer as described in previous study [23]. The DHE dye probe is oxidized to ethidium in the presence of superoxides. The fluorescence intensity was measured using a fluorescent plate reader at an extension wavelength of 520 nm and an emission wavelength of 620 nm. The values are represented as means ± SD of three sets of experiments. The percentages of cells with ROS were measured using a BD FACSCanto II flow cytometer machine (BD Biosciences).

### Statistical analysis

Experimental values were expressed as the means ± standard deviation (SD) of the number of experiments indicated in the legends. The analysis of variance (ANOVA) was performed using GraphPad Prism 5 software. Statistical significance was defined when P<0.05.

## Results and Discussion

### MTT cell viability assay

The cytotoxic effect of the solvents on cell viability was determined by MTT assay on OKF6, MCF-7, PC3, A375, HepG2, HT29 and H413 cells. The toxicity profile of synthesized DESs was assessed by comparing them with their individual components and their aqueous solutions, as illustrated in Table 1. The aqueous solutions were prepared using the same concentration of each component, in the DES, separately dissolved in distilled water.

**Table 1:** Numbering of DESs, their individual constituents and aqueous solutions.

**DES**	**Numbers**
ChCl:Gl	1_DES_
ChCl	2
Gl	3
ChCl:Glaq	4
ChCl:EG	5_DES_
ChCl	6
EG	7
ChCl:EGaq	8
ChCl:U	9_DES_
ChCl	10
U	11
ChCl:U	12
ChCl:TEG	13_DES_
ChCl	14
TEG	15
ChCl:TEGaq	16

Table 2 shows the IC_50_ values (the concentration of drug necessary to induce 50% inhibition on cell viability) for the solvents on studied cell lines. The solvents indicated relatively high cytotoxicity on the tested cell lines. It was found that the cell toxicity is dependent on DES composition, viscosity and concentration. In general, all cell lines are susceptible to DESs toxicity. Hence, the viability of cell lines is very limited, as stated in Table 2. In order to recognize whether this cytotoxicity is caused by one component or both components, they were tested using the same concentration of each component separately dissolved in distilled water. The HBDs and the salt used in preparing DESs showed a certain level of cytotoxicity in the aqueous solutions, corresponding to their concentrations in DESs. Based on this, the studied DESs (1_DES_,5_DES_,9_DES_,13_DES_) were found to have higher and/or less potent cytotoxicity than their individual components, indicating a synergistic effect after their mixing. This is in agreement with previous reported results for ammonium- and phosphonium-based DESs [6,12]. To verify whether this synergy is due to a normal mixing or whether there was a chemical change due to the formation of the DES, both substances were dissolved in the same amount of distilled water and then tested (Table 2).

**Table 2:** Cytotoxic activity of the solvents on various carcinoma and normal cells.

**Solvent**	**IC_50_ (μg/mL)**						
	**OKF6**	**MCF-7**	**PC3**	**A375**	**HepG2**	**HT29**	**H413**
1_DES_	47.26 ± 3.82	21.86 ± 2.54	30.65 ± 2.82	18.07 ± 1.62	36.08 ± 4.27	28.44 ± 3.28	54.67 ± 8.33
2	26.53 ± 1.97	8.95 ± 0.98	11.94 ± 1.35	7.584 ± 2.31	14.26 ± 1.43	13.41 ± 1.73	19.61 ± 4.19
3	129.31 ± 8.23	74.73 ± 6.67	42.38 ± 3.96	67.65 ± 6.83	68.85 ± 11.74	63.80 ± 7.23	101.02 ± 18.54
4	116.28 ± 11.27	41.38 ± 3.42	41.44 ± 2.19	59.00 ± 3.92	88.65 ± 9.54	81.17 ± 12.52	124.50 ± 14.82
5_DES_	69.71 ± 4.36	27.02 ± 1.31	32.88 ± 5.82	35.23 ± 4.40	24.74 ± 3.82	30.54 ± 3.68	56.60 ± 5.79
6	24.58 ± 2.81	13.05 ± 2.09	13.60 ± 2.94	8.64 ± 1.75	12.69 ± 1.63	11.79 ± 0.43	21.84 ± 2.65
7	75.62 ± 6.33	53.91 ± 6.17	32.48 ± 1.06	40.59 ± 5.27	44.33 ± 5.86	43.72 ± 3.97	62.29 ± 8.94
8	97.46 ± 5.57	32.90 ± 3.19	67.01 ± 4.71	26.37 ± 3.11	70.36 ± 8.21	56.30 ± 2.19	108.0 ± 15.29
9_DES_	81.93 ± 6.83	29.37 ± 4.83	27.78 ± 3.92	59.61 ± 8.28	37.71 ± 5.32	36.21 ± 4.98	68.02 ± 5.85
10	32.49 ± 3.41	12.51 ± 0.71	28.88 ± 2.64	10.54 ± 0.34	25.99 ±1.85	18.75 ± 1.65	38.41 ± 4.03
11	28.22 ± 1.05	8.96 ± 2.29	23.09 ± 5.22	11.21 ± 1.38	24.23 ± 4.02	13.93 ± 2.16	20.22 ± 1.69
12	41.55 ± 3.76	15.32 ± 3.66	24.15 ± 1.53	16.41 ± 2.74	18.83 ± 2.75	12.86 ± 0.27	38.08 ± 5.71
13_DES_	34.38 ± 2.19	16.09 ± 1.23	20.32 ± 2.34	12.29 ± 3.07	18.07 ± 3.19	17.42 ± 2.55	19.29 ± 2.42
14	21.74 ± 1.04	12.23 ± 0.39	10.18 ± 0.95	12.75 ± 1.23	14.60 ± 2.53	8.939 ± 1.43	21.49 ± 0.57
15	29.78 ± 1.45	18.27 ± 2.15	23.11 ± 1.32	14.62 ± 0.64	33.87 ± 4.94	20.23 ± 2.75	32.14 ± 3.10
16	32. 64 ± 2.73	21.77 ±1.97	17.20 ± 0.54	19.42 ± 3.18	29.67 ± 3.25	22.26 ± 0.46	35.72 ± 6.29

Table 2 shows that IC_50_ of DESs (1_DES_, 5_DES_, 13_DES_) is less than the aqueous solutions of both dissolved components 4, 8, 12, 16 (except in 5_DES_ for AT375 and 13_DES_ for OKF6, PC3). It must be noted that DESs are non-volatile compared to the aqueous solutions, which makes DESs more stable with less/without evaporation. However, Gutiérrez *et al*. (2010) demonstrated the hydration of the individual components of the DES: hydration results in the rupture of the hydrogen-bonded supramolecular complexes, and hence the DES would become a simple solution of the individual components [24]. Consequently, the special features of the DES in its pure state would vanish. However, the results are in disagreement, as the DESs that have been added to the media during the experiments acted differently in terms of IC_50_ evaluation compared to their aqueous solutions. For example, in 9_DES_ the IC_50_ is higher than its aqueous solution for all cell lines, indicating that after being synthesized DESs have their own character and are not a simple mixture of the two components. The chemical nature of DES components affects the viscosity of synthesized DES (e.g. the type of salt and HBD, organic salt/HBD molar ratio), water content and temperature [4,25].

Table 2 illustrates IC_50_ for the DESs 1_DES_, 5_DES_, 9_DES_ and 13_ES_, and follows the sequence of 13_DES_< 1_DES_< 5_DES_< 9_DES_ for OKF6, MCF-7, A375, HT29 and H413, respectively, while a slightly different sequence is noticeable for PC3 13_DES_< 9_DES_< 1_DES_< 5_DES_ and for HepG2 is 13_DES_< 5_DES_< 1_DES_< 9_DES_. This indicates that DES containing U as a HBD has the highest IC_50_ for all cells, followed by EG, Gl and TEG for OKF6, MCF-7, A375, HT29 and H413 cells. These data showed that the cytotoxicity of the DESs are cell-line dependent; for example, PC3 is more resistant to 5_DES_ than other cancer cell-lines. We noticed that most cancer cell-lines are resistant to 9_DES_ (except PC3). HBD of 9_DES_ is urea, which is an organic substance that is also produced in the body. Therefore, it is not toxic compared to other HBDs, since it is widely used as fertilizer.

Table 2 demonstrates that the investigated DESs inhibit cancer cell growth at certain dosages, indicating these results do not support the hypothesis that DESs are non-toxic solvents as shown by others [10,11,13,14]. A simple explanation for this would be that the hydrogen bonding between the HBD and the anion of the salt affects not only the physical properties but also the chemical structure of the mixture. This is in accordance with the fact that the type of HBD has a paramount effect on the toxicity of the corresponding DES [6]. It has been reported that the HBD, in its pure state, denatures the proteins in a living cell [26,27]. As a result, cell activity will be disrupted, and subsequently cell death may occur [12].

Gorke *et al*. (2008) investigated the activity of enzymes in the transesterification of ethyl valerate with butanol [28]. Enzymes were found to be stable in a ChCl/urea DES although they were poorly stable in an aqueous solution of urea (10 mol L^-1^) and ChCl (5 mol L^-1^). The stability was attributed to the hydrogen bond network of DES that lowered the DES components’ chemical reactivity towards enzymes.

To test the effect of DESs on the protein denaturation, a simple test was conducted using egg white (albumin). It was observed that no instantaneous denaturation took place after the addition of DESs to the albumin compared with addition of HCl. This result is in accordance with the study of Durand *et al*. (2012) in which they evaluated DESs as new media for Candida antarctica lipase B catalyzed reactions [29]. They reported that the immobilized Candida antarctica lipase B did not denaturate quickly in DESs containing U or Gl and that the stability in these DESs is sufficient to allow the reaction for several days.

Choi *et al*. (2011) proposed that natural DESs (NADESs) could be the missing link in understanding cellular metabolism and physiology explaining mechanisms and phenomena that are otherwise difficult to understand, such as the biosynthesis of non-water soluble small molecules and macromolecules [30]. Finally, they suggested that water and lipids are indeed not the only solvents present in living organisms.

Different cytotoxicity was reported for ILs depending on their structure. Some imidazolium-based ILs were found to be more toxic to human tumor cell line HeLa than conventional solvents [31–33]. IC_50_ of didecyldimethylammonium saccharinate [DDA][Sac] on HepG2 was 4.80 μM [31]. Muhammad *et al*. (2012) reported the IC_50_ of a series of choline carboxylate-based ILs on MCF-7 with a range of 10.5–16.0 μM, which is rather lower compared with the examined DESs [34]. In contrast, IC_50_ on MCF-7 and HepG2 was much higher for 13_DES_, 9_DES_, 5_DES_ and 1_DES_, at 47.99, 189.45, 169.89, 99.78 μM (on MCF-7) and 53.9, 243.25, 155.55, 164.68 μM (on HepG2) respectively. It is noteworthy that 13_DES_ has the lowest IC_50_ among DESs. The high difference between the IC_50_ of ILs and DESs is probably due to the complicated synthesis procedure, which usually requires toxic chemicals and agents like halides to synthesize ILs. On the other hand, DESs can be simply prepared by mixing components at a certain temperature.

To avoid the biohazardous side-effects of the DESs, it is necessary to investigate their selectivity in order to verify that non-target cells are not/less affected in order to achieve the optimal therapeutic effect. Hence, the cytotoxicity of the DESs on cancer cells was compared with OKF6 as a non-target cell line. The selectivity index of the solvents is presented in Table 3. The selectivity index values were varied (i.e.>2 or ≤2). 1_DES_ was selective against MCF-7 and A375, 5_DES_ was selective against MCF-7, PC3, HepG2 and HT29, 9_DES_ was selective against MCF-7, PC3, HepG2 and HT29, and 13_DES_ was selective against MCF-7 and A375. Nevertheless, further investigation is highly recommended to find other types of DESs with better selectivity index. It is noteworthy that the cytotoxicity of some DESs is also low as given by selectivity index ≤ 1 in the cell lines (Table 3). Other compounds such as piperidinyl-diethylstilbestrol, 4-Hydroxy tamoxifen and alisiaquinol have also previously been reported to have a selectivity index less than 2 [35,36].

**Table 3:** Selectivity index of the DESs for cancer cells compared with human oral keratinocyte cell line (OKF6).

**Solvent**	**Selectivity Index**					
	**MCF-7**	**PC3**	**A375**	**HepG2**	**HT29**	**H413**
**1**_DES_	2.162	1.542	2.615	1.309	1.662	0.864
**5**_DES_	2.579	2.120	1.979	2.812	2.282	1.232
**9**_DES_	2.789	2.949	1.374	2.172	2.263	1.204
**13**_DES_	2.137	1.692	2.797	1.902	1.974	1.782

**Fig 2:**
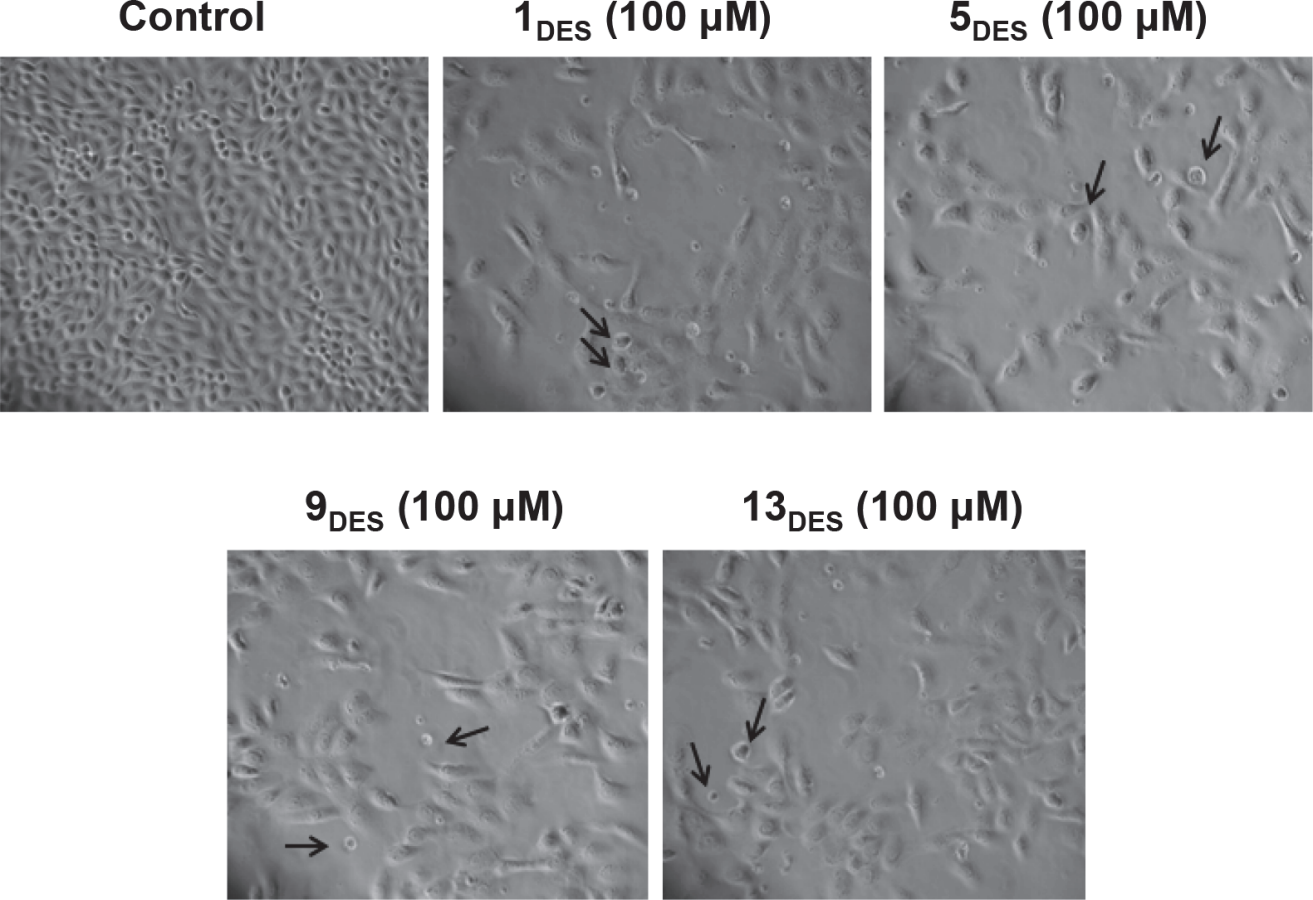
Morphologic alterations of MCF-7 cells treated with the DESs. Cells were treated with IC_50_ dose of each solvent for 24 h and their morphology was analyzed using light microscopy. (Arrow showing shrink or apoptotic cells)

**Fig 3:**
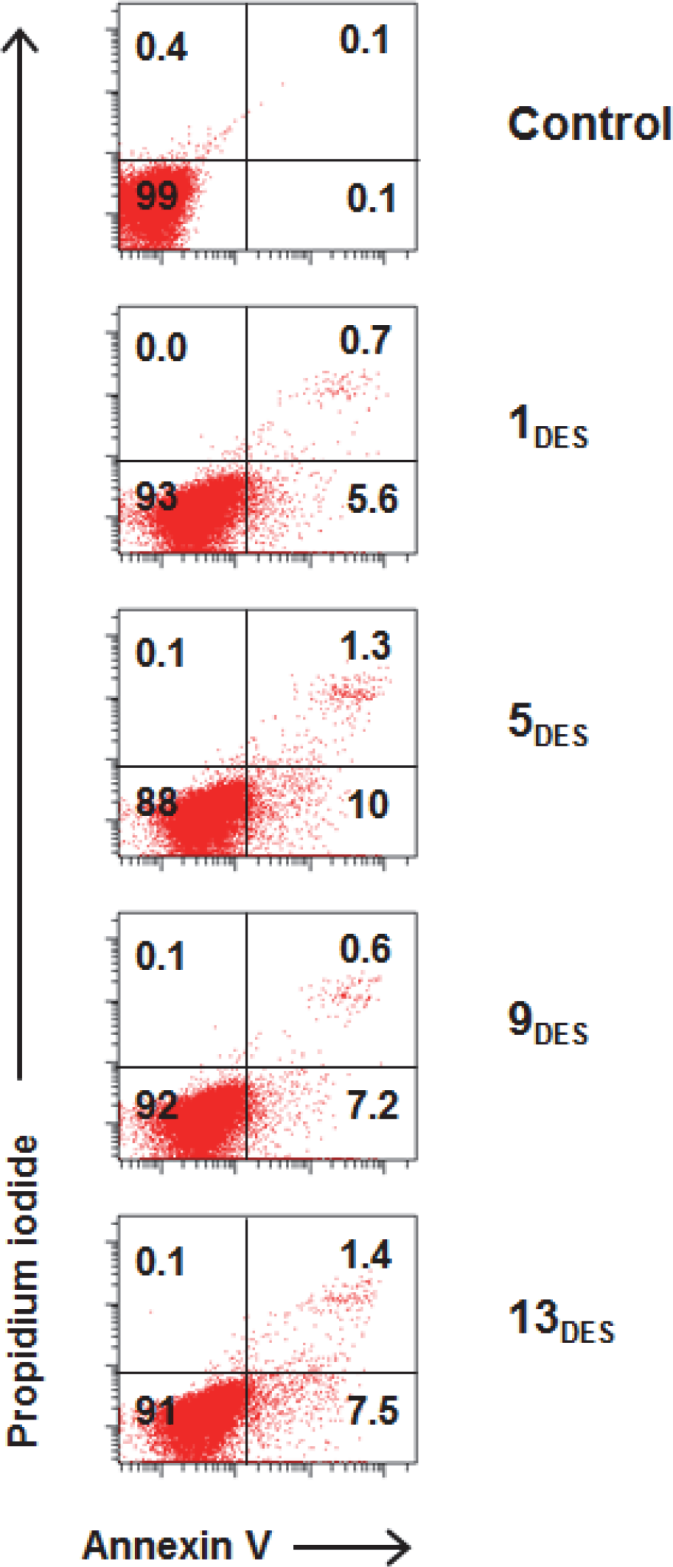
DESs induce cell apoptosis. **Apoptotic assay was examined in MCF-7 cells treated with 100 μg/mL DESs for 48 h.** Cells were stained with annexin V and propidium iodide (PI) to determine the percentages of live cells (annexin V+ PI+), early apoptotic cells (annexin V+ PI-) and late-stage apoptotic cells (annexin V+ PI+).

To demonstrate the cytotoxic effect, MCF-7 was treated with IC_50_ dosages of each DESs for 24 h and examined the morphological changes using light microscopy. As shown in Fig. 2, the number of MCF-7 cells in treated group was less confluent compared to control. In addition, some cells shrink and appeared apoptotic as indicated by the arrows. To further examine if DESs treatment induces cell apoptosis, cells were stained with annexin V and propidium iodide (PI) before analysed with a flow cytometer. The DESs treated cells demonstrated increased percentages of early (annexin V+ PI-) and late apoptotic/death (annexin V+ PI+) cells, compared to control (Fig. 3).

**Fig 4:**
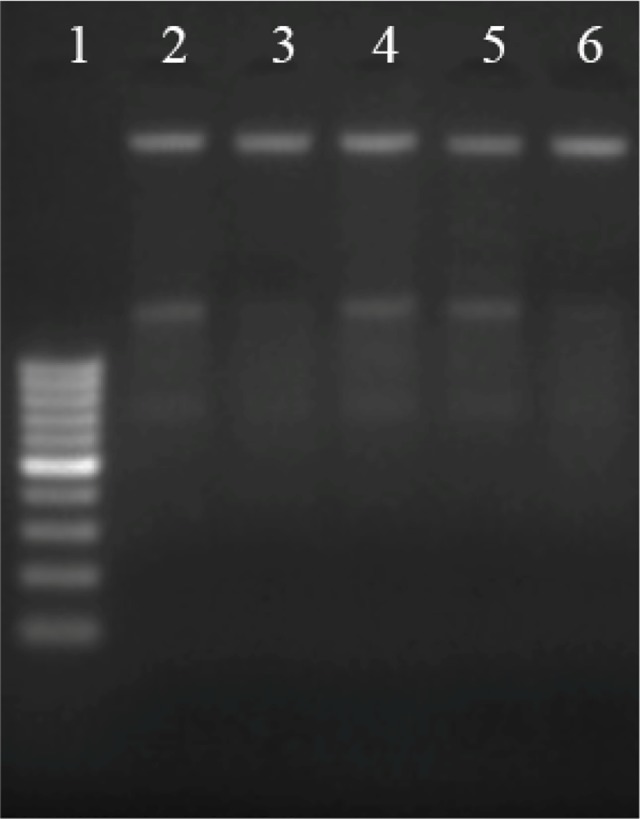
DNA fragmentation analysis of MCF-7 cells treated with DESs, using agarose gel electrophoresis. Lane 1, 100bp. DNA marker; Lane 2, untreated; Lane 3–6, treated with 1_DES_, 5_DES_, 9_DES_ and 13_DES_, respectively.

Next, DNA was harvested from control and DES-treated MCF-7 cells to evaluate whether DESs have any effect on the integrity of DNA. Agarose gel electrophoresis results show that no DNA fragmentation is observable in the treated group (Fig. 4). This implies that the cell death caused by the tested DESs might be through another mechanism, rather than apoptotic DNA fragmentation.

**Fig 5:**
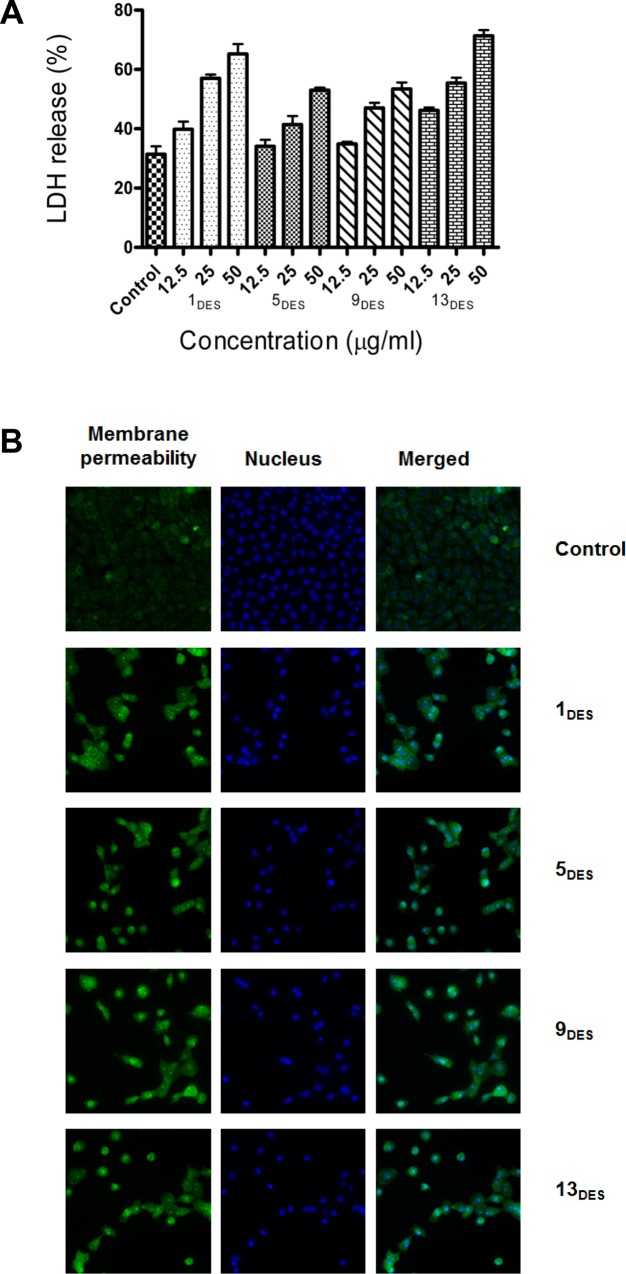
DESs treatment increase LDH release and membrane permeability. (A) LDH% release by MCF-7 cells treated with various dosages of DESs. After 48 h treatment, the released LDH were quantified by a coupled enzymatic reaction, and measured using a fluorescence plate reader. (B) MCF-7 cells treated with LC_50_ dosages of DESs for 24 h. Cells were fixed and stained with membrane permeability dye (green) and nucleus was stained with Hoechst 333258 dye (blue).

Lactate dehydrogenase (LDH) is a cytosolic enzyme present in many different types of cells. When the plasma membrane is damaged, LDH is released into cell culture media. The released LDH can be quantified by a coupled enzymatic reaction that indicates the cytotoxicity of the solvents. The cytotoxicity of the solvents by LDH release assay was determined on MCF-7 cells treated with different concentrations of the solvents for 48 h. LDH release in the medium was due to the loss of membrane integrity either due to apoptosis or necrosis. All DESs cause cytotoxicity in a dose dependent manner; with 13_DES_ exhibiting the most significant increase in LDH-release level (~45–70%) in comparison to control cells (Fig. 5A).

We hypothesized that the increased LDH release might indicate compromised plasma membrane. To examine this, MCF-7 cells was treated with DESs for 24 h. Then, live cells were incubated with a cell membrane permeability dye. Cells were fixed and visualized with Cellomics high content screening. As shown in Fig. 5B, control cells with intact plasma membrane resulted in less permeability of the dye into the cytosol. In contrast, DESs treated cells stained strongly with the permeability dye, indicating damaged plasma membrane.

**Fig 6:**
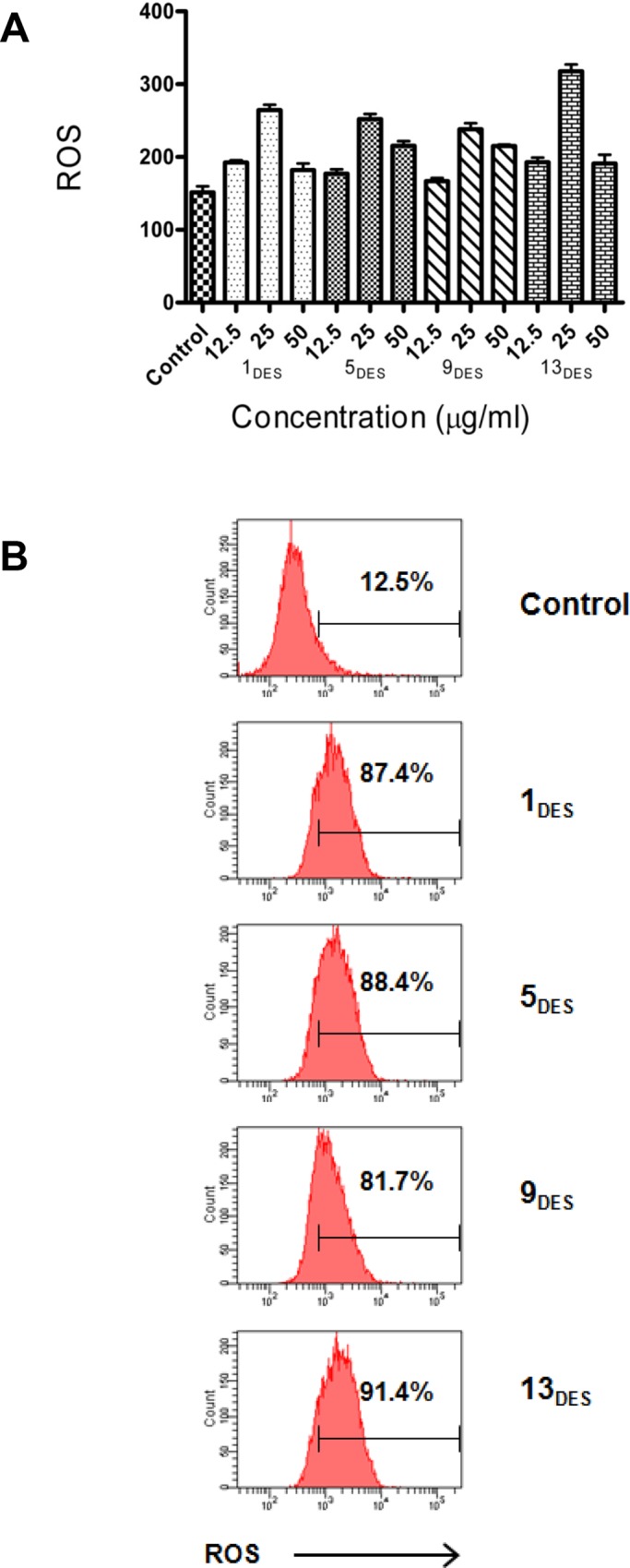
ROS level in MCF-7 cells treated with various concentrations of DESs. MCF-7 cells were pretreated with DESs for 24 h and stained with DHE dye to determine the ROS production. The fluorescent intensities of DHE oxidization by ROS were measured using a (A) fluorescence microplate reader and (B) MCF-7 cells treated with 50 μg/mL DESs for 24 h were analyzed by a flow cytometer machine.

MCF-7 cells were pretreated with the solvents for 24 h and stained with DHE dye to determine the influence of DES exposure on ROS production. The fluorescent intensities of DHE oxidization by ROS were measured using a fluorescence microplate reader and flow cytometer. As shown in Fig. 6, exposure to the four DESs causes an increase in the ROS level of the treated MCF-7 cells. These changes are more significant at the concentrations close to the IC_50_ of the solvents. The highest oxidative stress was exerted by 13_DES_, in which the fluorescent intensity of the cells treated with 25 μg/mL was two times higher than that of the control cells. This increment in ROS can be ascribed to the burden of DESs on the antioxidant enzyme superoxide dismutase (SOD). SOD is responsible for clearing off the free radicals generated by the ROS. Increased ROS production by DESs can overwhelm the SOD functions by inducing apoptosis in targeted cells. This hypothesis is to some extent supported by Dai *et al*. (2013), who reported that mixtures of many abundant primary metabolites from all kinds of organisms can form NADESs when mixed in adequate ratios [1]. Various materials were found to be soluble in NADES, such as some non-water soluble bioactive natural products, gluten, starch, DNA, proteins and polysaccharides. The high solubilizing capacity is related to their supramolecular structure and broad polarity range. The existence of NADES in plants and their properties indicate that NADES might be involved in the biosynthesis and storage of various non-water soluble metabolites in cells and imply the role of NADES in protecting organisms from extreme conditions.

Overall, the relatively high cytotoxicity and low selectivity index of the solvents demonstrated considerable risks associated with their applications. The results also indicated a significant role for the HBD on the cytotoxic activity of the DES. However, further investigations may contribute to clarify such effects of HBD on the activity of DES.

### LD_50_

Table 4 illustrates the LD_50_ of the studied DESs with their individual components. In general, all DESs are relatively toxic with LD_50_ values of 6.39±0.53, 5.33±0.49, and 5.31±0.62 for 1_DES_, 5_DES_ and 13_DES_, respectively. This is in accordance with the *in vitro* cytotoxicity results and in disagreement with previous studies that showed DESs are non-toxic to the bacteria, brine shrimp and other microorganisms [6,12,37]. Moreover, it can be noticed that the LD_50_ of all DESs is less than their individual components (i.e. pure Gl, EG, U, TEG and ChCl) indicating the synergistic toxicity effect of these mixtures after being prepared. Of note, 9_DES_ caused an immediate death to animals (LD_50_ could not be determined). In contrast, DES prepared from ChCl:U with a molar ratio of 1:2 (denoted as 9_DES_^/^) showed LD_50_ at 5.64 g/kg, compared to a molar ratio of 1:3 for 9_DES_. Hence, the acute toxicity effect can be ascribed to the molarity of this DES. This clearly shows that the molar ratio of DESs plays a primary role in the toxicity profile of these mixtures. The LD_50_ followed the order of 1_DES_ > 9_DES_^/^ >5_DES_ > 13_DES_.

**Table 4:** The LD_50_ values for DESs and their components.

**Solvents**	**LD_50_ (g/kg)**
**1**_DES_	6.39±0.53
**5**_DES_	5.33±0.49
**9**_DES_	toxic
9_DES_^/^	5.64±0.36
**13**_DES_	5.31± 0.62
Pure Gl	20.6±2.16
Pure EG	9.71±1.95
Pure TEG	16.98±2.04
Pure U	>20
Pure ChCl	>20

Table 5 shows the blood test results of mice orally administrated with DESs. Most of the parameters for renal function test (sodium, potassium, chloride and urea concentrations) were mildly elevated in the DESs-treated mice, compared to the normal range. In the liver function test, serum alanine aminotransferase (ALT) and alkaline phosphatase (ALP) showed normal concentrations. However, the serum aspartate transaminase (AST) levels were substantially increased to 5.7 to 9.5 folds above the normal range in the DESs-treated mice. The increase of AST:ALT ratio suggested that DESs may cause hepatocellular pattern of liver injury.

**Table 5:** Blood test of mice tested.

**Clinical Chemistry**	**1_DES_**	**5_DES_**	**9_DES_^/^**	**13_DES_**	**Unit**	**Ref. Range**	
**Renal Function Test**							
Sodium	146± 1.41	149.5± 3.54	149±2.52	146.5±2.12	mmol/L	136	-145
potassium	8.2± 0.28	7.65± 1.06	9.1±0.89	7.75± 0.78	mmol/L	3.6	-5.2
Chloride	112.5± 0.71	112.5± 3.54	111±3.11	112±2.83	mmol/L	100	-108
Carbon dioxide	14.75± 0.71	13.3± 5.09	17.6±3.78	14±2.263	mmol/L	21	-30
Anion Gap	27±0.00	31.5± 6.36	30±4.95	28.5±3.54	mmol/L	10	-20
Urea	8.75± 0.21	5.85± 0.78	9.1±0.46	9.05±0.21	mmol/L	2.5	-6.4
Creatinine	18± 0	16± 2.83	10±1.63	16±0.00	umol/L		
**Liver Function Test**							
Total Protien	59± 5.66	59± 2.83	46±3.98	61±5.66	g/L	64	-82
Albumin	15± 0.00	12.5± 0.71	12±0.43	16±0	g/L	35	-50
Globuline	44± 5.66	46.5± 3.54	34±4.47	45±5.66	g/L	23	-35
Total Bilirubin	<2	3±0.00	2±0.00	<2	umol/L	3	-17
Conjugated Bilirubin	<1	<1	<1	<1	umol/L	0	-3
Alkaline Phosphatase (ALP)	58± 0	50.5± 7.78	40±6.08	63±5.66	IU/L	50	-136
Alanine Aminotransferase (ALT)	54.5± 0.71	54.5± 17.68	32±2.92	48.5± 4.95	IU/L	12	-78
Aspartate transaminase (AST)	275± 5.66	309.5± 62.93	213±50.11	265± 72.13	IU/L	15	-37
G-Glutamyl Transferase	<3	5± 1.56	<3	<3	IU/L	15	-85
**Lipid Profile**							
Triglyceride	1.3± 0.00	0.7± 0.0	0.8±0.12	1.55± 0.35	mmol/L	<1.7	-
Total Cholestrol	1.45± 0.01	2.25± 0.36	2.9±.56	2.35± 0.92	mmol/L	<5.2	-
HDL Cholestrol	1.54± 0.14	2.11± 0.71	2.82±0.81	2.335± 0.96	mmol/L	<1.1	-

### ORAC anti-oxidant activity assay

ORAC assay has been widely used for quantifying activity via area under curve (AUC) technique and for measuring antioxidant capacity, as it is the only assay that involves the use of peroxyl radical as pro-oxidant [38]. In this work, quercetin was used as the standard for comparison of antioxidant activity. The performed assay showed that the DESs exhibit very low antioxidant activity in comparison to quercetin (Table 6), which indicates that these DESs cannot serve as radical scavengers.

**Table 6:** Antioxidant capacity of the DESs by ORAC method.

**Solvent**	**net AUC**	**mM of Trolox per 100μg/mL**	**μM of Trolox per 100μg/mL**
1 _DES_	399354	0.0001346	0.135
5 _DES_	369172	0.0001244	0.124
9 _DES_	157560	0.0000531	0.053
13 _DES_	411860	0.0001388	0.139
Quercetin	6353346	0.0214108	21.411

## Conclusion

The combined *in vitro* and *in vivo* toxicity profiles of ammonium-based DESs have been investigated and reported for the first time in this study. Results showed that the cytotoxicity and selectivity can be influenced by the composition of DESs by varying the salt/HBD combination and molar ratio. Further studies are needed to fine-tune the property of DESs to reduce toxicity effect before being implemented as potential therapeutic agents (e.g. alternative drugs or drugs vehicles).
